# Parent perspectives on school food allergy policy

**DOI:** 10.1186/s12887-018-1135-6

**Published:** 2018-05-12

**Authors:** S. Shahzad Mustafa, Anne F. Russell, Olga Kagan, Lauren M. Kao, Diane V. Houdek, Bridget M. Smith, Julie Wang, Ruchi S. Gupta

**Affiliations:** 1 Rochester Regional Health, Rochester, NY USA; 2 University of Rochester School of Medicine and Dentistry, Rochester, NY USA; 3 Food Allergy & Anaphylaxis Michigan Association, Ann Arbor, MI USA; 4 Molloy College, Rockville Centre, New York, USA; 5 Northwestern University Feinberg School of Medicine, Chicago, IL USA; 6 Center of Innovation for Complex Chronic Healthcare, Edward J. Hines, Jr. Veterans Affairs Hospital, Hines, IL USA; 7 Icahn School of Medicine at Mount Sinai, New York, NY USA; 8 Ann & Robert H. Lurie Children’s Hospital of Chicago, Chicago, IL USA; 9 Center for Community Health, Northwestern University Feinberg School of Medicine, 750 N Lake Shore Dr., 6th Floor, Chicago, IL 60611 USA

**Keywords:** Anaphylaxis, Food allergy, School, Policy, Epinephrine, Food labeling

## Abstract

**Background:**

Food allergy affects up to 8% of children in the U.S. There is minimal research to date on food allergy policies that are currently in place in schools and the opinions of parents of children with food allergy on the effectiveness of or need for these policies.

**Methods:**

An electronic survey was disseminated to parents of children with food allergy. Frequencies were calculated to describe respondent characteristics and responses. Chi-square tests were performed to examine associations between school and child characteristics and outcomes.

**Results:**

Of the 289 parent respondents, 27.4% were unsure or felt school was unsafe for their child with food allergy. While the majority felt that the polices in their child’s school were helpful, most also believed that implementation of additional polices was necessary, including availability of stock epinephrine (94.2%), lunch menus with allergen information (86%), ingredient labels on food items (81%), and direct food allergy education for students (86%). There were significant differences in school food allergy policy depending on the age of the student body, private versus public school, and geographic location.

**Conclusions:**

While most schools reportedly have one or more food allergy policies in place, many parents have concerns over the safety of their child at school and feel that additional policies are necessary to improve the safety of the school environment for children with food allergy. The availability of stock epinephrine, improved allergen labeling of food and menus and increased food allergy education may be key policy areas on which to focus.

## Background

Schools must practice effective anaphylaxis prevention and preparedness strategies as increasing prevalence rates of childhood food allergy have created a significant public health issue. It is estimated that nearly 6 million U.S. children, or 8%, now have some form of IgE-mediated food allergy, with 30% of food allergic children reporting multiple food allergies [1, 2]. Despite ongoing research into potential therapeutic options, current management is based on strict avoidance of known food allergens and appropriate response to accidental exposures [3]. Although fatalities from food allergic reactions in school are rare, food is often ubiquitous throughout the school setting, thus creating risks for accidental exposures in schools that may have varying levels of anaphylaxis preparedness. Since most children spend up to half their waking hours at school, management of food allergy in the school setting is an important issue.

The U.S. Department of Education has not established policy recommendations related to food allergy. While voluntary guidelines for the management of food allergy in schools have been developed by the Center for Disease Control, there remains significant heterogeneity in school preparedness for food allergic reactions and there is no consensus on which preventative policies work best for improving food allergy safety in schools [4, 5]. The vast majority of schools have at least one student with food allergy, and one survey showed that 67% of schools had made at least one accommodation for children with food allergy (peanut-free tables, peanut ban in classrooms, or alternative meals) [6]. However, approximately 18% of children with food allergies have experienced an allergic reaction while at school and over 10% of 5683 schools reported at least one anaphylactic event during the 2013-2014 academic year [6–9]. Therefore, parents and students may have significant concerns regarding the potential for food-induced allergic reactions at school as well as the ability of school staff to effectively manage anaphylaxis.

To date, little research has been conducted examining current food allergy policies in US school systems and even less on the opinions of parents of children with food allergy regarding those policies and their utility. Therefore, the goal of this study was to survey parents of children with food allergy to determine the food allergy policies that they are aware of in their child’s school and their opinion on the effectiveness of or need for these policies. With a better understanding of school policies, stronger recommendations for food allergy management in schools may be made to promote a safe and conducive learning environment for all children.

## Methods

An initial survey was developed with the goal of understanding school policies related to food allergy. The research domains were selected based on a review of relevant literature and included family demographics (age, gender, race, grade, allergic reaction experience, etc.), awareness of current school food allergy policies; acceptability, effectiveness, and feasibility of current policies; and desired school food allergy policies. Following initial survey development, cognitive interviews were conducted with a subset of parents (*n* = 5) recruited through food allergy support groups in order to refine the survey questions. The final survey tool consisted of 105 multiple-part, multiple-choice, and open-ended response questions with skip logic, which required approximately 10 min to complete. It is available upon request. Parents were asked to respond for only one of their children with food allergy. Severe allergic reaction was not defined for respondents. Respondents who reported that a given policy was in place at their child’s school were asked if the policy was helpful, while those who reported that a given policy was not in place or were unsure were asked if they felt that the policy was needed. No incentive was offered for participation in the study. REDCap (Research Electronic Data Capture, Vanderbilt University) hosted at Northwestern University was used to administer the online survey [10].

### Selection of subjects

Parents were recruited for participation through the Allergy and Asthma Network (AAN) and Mothers of Children Having Allergies (MOCHA) listservs. The target population included parents of children with food allergies in grades 1-12. Eligible participants were invited to participate via an email containing a link to complete the survey. Consent was obtained before any participant could access the survey. The process of obtaining informed consent followed all applicable requirements. The survey was conducted from August 2016 through January 2017. No identifying information was collected and all responses were kept confidential on secure servers at Northwestern University. The study was deemed exempt by Northwestern University’s Institutional Review Board.

### Statistical analyses

All statistical analyses were performed in Stata 14.0 statistical software (Stata Corp, College Station, TX). Frequencies were calculated to describe respondent characteristics and responses. Responses to the policy questions were dichotomized into two categories: yes and no/unsure. Missing responses were coded with “no/unsure.” For demographic variables, missing variables were coded as “other” or were included in a separate category. To examine associations between school and respondent characteristics and outcomes, chi-square tests were performed.

## Results

### Survey respondents

Of 290 completed surveys, one was eliminated due to the child not meeting grade level eligibility criteria, resulting in a final sample of 289 responses included in the final analyses. The majority of respondents were mothers (91.3%), had a household income of more than $100,000 (55.4%), and held a college degree or higher (85.5%) (Table 1). Most respondents lived in the Northeast (35.3%) or Midwest (36.3%). The majority of children were male (63.4%) and Caucasian (77.9%). 70% of the children were in elementary grades (grades 1-5), and most attended a public school (78.6%). The most common food allergens were peanut (76.1%) and tree nuts (77.2%) and the most common comorbid conditions were allergic rhinitis (78.9%), asthma (55.7%), and atopic dermatitis (49.8%). While 81% of respondents reported that their child had experienced at least one severe reaction in their lifetime, 82% reported that their child had never experienced a severe reaction in the school setting.

**Table 1:** Respondent Demographics

Characteristic	Frequency, N (%)
Total Number of Respondents	289
Child’s gender	
Male	176 (63.4)
Female	99 (34.3)
Other/Would not like to report/Missing (missing = 12)	14 (4.8)
Child’s grade	
Elementary (1-5)	182 (70.0)
Middle (6-8)	46 (15.9)
High school (9-12)	35 (12.1)
Missing	26 (9.0)
Type of school	
Public	227 (78.6)
Private	16 (5.5)
Religious School	29 (10.0)
Other	17 (5.9)
How many severe reactions has your child had in his or her lifetime	
0	55 (19.0)
1	88 (30.5)
> 1	146 (50.5)
How many severe reactions has your child had in school in his or her lifetime	
0	237 (82.0)
1	35 (12.1)
> 1	17 (5.9)
How many severe reactions has your child experienced in school in the past year	
0	265 (91.7)
1	19 (6.6)
> 1	5 (1.7)
To which foods is your child allergic	
Peanut	220 (76.1)
Tree nut	223 (77.2)
Fin fish	21 (7.3)
Shellfish	41 (14.2)
Milk	106 (36.7)
Egg	97 (33.56
Soy	27 (9.3)
Wheat	26 (9.0)
Sesame	55 (19.0)
Other	69 (23.9)
Ethnicity (Hispanic or Latino)	
Yes	12 (4.2)
No	254 (87.9)
Unknown	20 (6.9)
Unspecified	3 (1.0)
Race	
Black/African American	9 (3.1)
White	225 (77.9)
Asian	11 (3.8)
American Indian/Alaska Native	3 (1.0))
Native Hawaiian or other Pacific Islander	0
Other	16 (5.5)
Unknown	25 (8.7)
Child’s comorbid conditions	
Asthma	161 (55.7)
Eczema	144 (49.8)
Seasonal allergy	228 (78.9)
Indoor allergy	98 (33.9)
Pet allergy	153 (52.9)
Insect allergy	25 (8.7)
Medication allergy	61 (21.0)
None	15 (5.2)
Other	9 (3.1)
Respondent’s relationship to child	
Mother	263 (91.3)
Father	9 (3.3)
Grandparent	0
Other	3 (1.0)
Unknown	14 (4.8)
Highest level of education	
Some secondary school (9th grade and above)	2 (0.7)
High school graduate or GED	3 (1.0)
Some college	20 (6.9)
College degree	144 49.8)
Master’s degree	80 (27.7)
Doctoral degree	23 (8.0)
Unknown	17 (5.9)
Income	
< $50,000	18 (6.2)
$50,000-$74,999	24 (8.3)
$75,000-$99,999	39 (13.5)
$100,000 or higher	160 (55.4)
Unknown	48 (16.6)
Region	
Northeast	102 (35.3)
Midwest	105 (36.3)
South	31 (10.7)
West	20 (6.9)
Unknown	31 (10.7)

### Food allergy and anaphylaxis policies

Of the 289 parent respondents, 18.7% felt that school was unsafe for their food allergic child, and an additional 8.7% were unsure about their child’s safety while at school (Table 2). Regarding the availability of epinephrine, approximately half of parents reported that their child was allowed to self-carry their epinephrine (57.8%), that their child’s epinephrine was readily available in the classroom (50.2%), and that their child’s school had non-student-specific stock epinephrine available (53.7%). Stock epinephrine was less frequently reported to be available on field trips (36%), after school (10%) or to travel with groups to off-site after-school events (11.4%). However, nearly one-quarter (24.2%) of parents did not know if their child’s school had stock epinephrine available, with 28.6% reporting uncertainty about epinephrine policies related to field trips and 54.3% reporting uncertainty about epinephrine policies relating to after-school activities). The majority of parents who reported that policies related to epinephrine were in place felt that these policies were helpful (87.9-96.7% depending on the policy). Similarly, nearly all parents who reported that epinephrine policies were not in place felt that such policies were needed (81.2-92.2% depending on the policy). In the lunchroom, the policies most frequently reported to be in place were designated areas in the lunchroom for students with food allergy (63.4%) and clear cleaning procedures (55.4%). Parents were least likely to report that menus with allergen information were available to them (34.6%) and that food items were labeled with allergen information (12.5%). The majority of parents felt that the lunchroom policies in place in their child’s school were helpful (95-91.7%) or that such policies were needed (91.2-80.6%), with the exception of having designated lunch areas for students with food allergy (helpful = 69.6%, needed = 32.1%).

**Table 2:** School Food Allergy Policies

Policy	Yes	No	Unsure
	*N* = 289		
School is a generally safe environment	210 (72.7)	54 (18.7)	25 (8.7)
Epinephrine Policies			
Emergency (stock) epinephrine is available	155 (53.7)	64 (22.2)	70 (24.2)
Policy is Helpful (if responded “Yes”)/Needed (if responded “No”)	146 (94.2)	59 (92.2)	
Children are able to carry their medications	167 (57.8)	86 (29.8)	36 (12.5)
Policy is Helpful/Needed	156 (93.4)	47 (54.7)	
Child’s epinephrine is readily available in the classroom	145 (50.2)	129 (44.6)	15 (5.2)
Policy is Helpful/Needed	–	–	
Emergency (stock) epinephrine available on all school field trips (*N* = 287)	104 (36.3)	101 (35.2)	82 (28.6)
Policy is Helpful/Needed	95 (91.4)	82 (81.2)	
Who carries epinephrine on field trips (if responded “Yes”)			
Teacher	60 (58.3)		
School-appointed chaperone	15 (14.6)		
School nurse	8 (7.8)		
Unsure	6 (5.8)		
Other	14 (13.6)		
Emergency (stock) epinephrine available for after-school activities	30 (10.4)	136 (47.1)	123 (42.6)
Policy is Helpful/Needed	29 (96.7)	119 (88.8)	
Who carries epinephrine during after-school activities (if responded “Yes”)			
Athletic trainer	1 (3.3)		
School nurse	3 (10.0)		
School staff	7 (23.3)		
Other	19 (63.3)		
Emergency (stock) epinephrine travels with every group	33 (11.4)	99 (34.3)	157 (54.3)
Policy is Helpful/Needed	29 (87.9)	82 (82.8)	
Who carries epinephrine during after-school activities (if responded “Yes”)			
Athletic trainer	1 (3.0)		
School nurse	4 (12.1)		
School staff	10 (30.3)		
Coach/teacher	9 (27.3)		
Other	11 (33.3)		
Lunchroom Policies			
Designated lunch areas for students with food allergies	184 (63.4)	78 (27)	27 (9.3)
Policy is Helpful/Needed	128 (69.6)	25 (32.1)	
School lunch menus with allergen information available	100 (34.6)	128 (44.3)	61 (21.1)
Policy is Helpful/Needed	93 (93)	110 (85.9)	
Food items are labeled with allergen information	36 (12.5)	144 (49.8)	109 (37.7)
Policy is Helpful/Needed	33 (91.7)	116 (80.6)	
Clear cleaning procedures in the lunchroom	160 (55.4)	34 (11.8)	95 (32.9)
Policy is Helpful/Needed	152 (95)	31 (91.2)	
Classroom Policies			
Snack policy in the classroom	178 (61.6)	98 (33.9)	13 (4.5)
Policy is Helpful/Needed	159 (89.3)	63 (64.3)	
Strict food guidelines for celebrations (holidays and birthdays) (N = 287)	153 (53.3)	120 (41.9)	14 (4.9)
Policy is Helpful/Needed	138 (90.2)	96 (80)	
What are the recommendations (if responded “Yes”)			
Food with a clear ingredient label is allowed	59 (38.6)		
No food is allowed	48 (31.4)		
Unsure	4 (2.6)		
Other	42 (27.5)		
Field Trip & After-School Policies			
When food is not provided by the school for field trips, all parents are provided with food guidelines	74 (25.6)	154 (53.3)	61 (21.1)
Policy is Helpful/Needed	58 (78.4)	104 (69.3)	
Strict food policies for after-school activities	23 (8.0)	161 (55.7)	105 (36.3)
Policy is Helpful/Needed	22 (95.7)	120 (74.5)	
Concessions are clearly labeled for food allergens	18 (6.2)	152 (52.6)	119 (41.2)
Policy is Helpful/Needed	18 (100)	133 (87.5)	
Food Allergy Education Policies			
Training and education for students	31 (10.7)	207 (71.6)	51 (17.7)
Policy is Helpful/Needed	28 (90.3)	177 (85.5)	
Educational materials in the lunchroom relating to food allergy	18 (6.2)	176 (60.9)	95 (32.9)
Policy is Helpful/Needed	14 (77.8)	136 (77.3)	
Educational materials in the classroom relating to food allergy (N = 287)	16 (5.6)	210 (73.2)	
Policy is Helpful/Needed	13 (81.3)	161 (76.7)	61 (21.3)
Transportation Policies			
Children take the school bus to/from school	129 (44.6)	155 (53.6)	5 (1.7)
Adult on school bus to/from school is trained on allergic reactions (if responded “Yes”)	48 (37.2)	33 (25.6)	48 (37.2)

In the classroom, a snack policy was reported to be in place by 61.6% of parents and strict food guidelines for celebrations were reported by 53.3%. These policies were deemed helpful by 89.3 and 90.2% of parents, respectively. While 80% of parents who reported that classroom celebration food guidelines were not in place felt they were needed, only 64.3% felt a classroom snack policy was needed.

Policies related to food allergy education and training were among the least-frequently reported. While 37.2% reported that an adult on their child’s bus was trained in the use of epinephrine, only 10.7% reported that food allergy education/training were available for students, 6.2% reported that lunchroom educational materials were available, and 5.6% reported that classroom educational materials were available. However, the majority of parents felt that such policies were helpful (90.3-77.8%) or needed (85.5-76.7%).

Policies related to field trips and after-school were also among those less frequently reported to be in place. One-quarter (25.6%) of parents reported that food guidelines were provided for field trips, 8.0% reported that strict food policies were in place for after-school activities, and 6.2% reported that concessions were labeled with allergen information. However, many parents reported being unsure of whether their child’s school had these policies (concessions labeled = 37.7%, after school food policies = 36.3%). The majority of parents felt that field trip and after-school policies were either helpful (100-78.4%) or needed (87.5-69.3%).

### Associations between food allergy policies and school characteristics

Significant associations were noted between several food allergy policies and student age (Table 3). As expected, more students were reported to be allowed to self-carry epinephrine in high school and middle school as compared to elementary school (91.4, 78.3, 47.3%, *p* < 0.01). Parents of elementary and middle school students reported that their child’s school had designated areas in the lunchroom (elementary = 72.5%, middle = 67.4%, high = 20.0%, *p* < 0.01), clear lunchroom cleaning procedures (elementary = 60.4%, middle = 65.3%, high = 20.0%, *p* < 0.01), and strict classroom policies for snacks (elementary = 69.8%, middle = 52.2%, high = 31.4%, *p* < 0.01) and celebrations (elementary = 56.6%, middle = 54.4%, high = 34.3%, *p* < 0.01) more frequently than parents of high school students.

**Table 3:** Unadjusted Association with Outcomes: Grade Level (Yes vs. No/Unsure/No Response)

Policy	Grade Level			
	Elementary	Middle	High	Unknown
	*N* = 182	*N* = 46	*N* = 35	*N* = 26
School is a generally safe environment	137 (75.3)	34 (73.9)	24 (68.6)	15 (57.7)
Epinephrine Policies				
Emergency (stock) epinephrine is available	95 (52.2)	28 (60.9)	20 (57.1)	12 (46.2)
Children are able to carry their medications	86 (47.3)	36 (78.3)	32 (91.4)	13 (50.0)**
Child’s epinephrine is readily available in the classroom	94 (51.7)	22 (47.8)	16 (45.7)	13 (50.0)
Emergency (stock) epinephrine available on all school field trips	63 (34.6)	20 (13.4)	9 (25.7)	12 (46.2)
Emergency (stock) epinephrine available for after-school activities	14 (7.7)	10 (21.7)	4 (11.4)	2 (7.7)
Emergency (stock) epinephrine travels with groups outside of school	18 (9.9)	6 (13.0)	5 (14.3)	4 (15.4)
Lunchroom Policies				
Designated lunch areas for students with food allergies	132 (72.5)	31 (67.4)	7 (20.0)	14 (53.9)**
School lunch menus with allergen information available	54 (29.7)	18 (39.1)	16 (45.7)	12 (46.2)
Food items are labeled with allergen information	24 (13.2)	4 (8.7)	5 (14.3)	3 ((11.5)
Clear cleaning procedures in the lunchroom	110 (60.4)	30 (65.2)	7 (20.0)	13 (50.0)**
Classroom-Specific Policies				
Snack policy in the classroom	127 (69.8)	24 (52.2)	11 (31.4)	16 (61.5)**
Strict food guidelines for celebrations (holidays and birthdays)	103 (56.6)	25 (54.4)	12 (34.3)	13 (50)**
Field Trip & After School Policies				
When food is not provided by the school for field trips, all parents are provided with food guidelines	52 (28.6)	10 (21.7)	5 (14.3)	7 (26.9)
Strict food policies for after-school activities	14 (7.7)	5 (6.5)	0 (0.0)	3 (11.5)
Concessions are clearly labeled for food allergens	11 (6.0)	4 (8.7)	0 (0.0)	3 (11.5)
Food Allergy Education Policies				
Training and education for students	22 (12.1)	5 (10.9)	2 (5.7)	2 (7.7)
Educational materials in the lunchroom relating to food allergy	8 (4.4)	5 (10.9)	2 (5.7)	3 (11.5)
Educational materials in the classroom relating to food allergy	9 (5.0)	2 (4.4)	2 (5.7)	3 (11.5)
Children take school bus to/from school	82 (45.1)	25 (54.4)	14 (40.0)	8 (30.8)
Adult on school bus is trained on allergic reactions	32 (39.0)	12 (48.0)	2 (14.3)	2 (25.0)

**p < 0.01, *p < 0.05

School type (public versus private) was also significantly associated with reported food allergy policies (Table 4). Parents of children attending public schools more frequently reported that their child’s school had a designated area in the lunchroom compared to private schools (67.4% vs 50%, *p* < 0.05). Parents of children attending private schools more frequently reported that their child’s epinephrine was available in the classroom (62.9% vs 46.7%, *p* < 0.05) and that strict food guidelines were in place for field trips when food was not provided by the school (38.7% vs 22%, *p* < 0.01). Finally, parents of children in private schools more frequently reported that students were provided with training and education on food allergy (17.7% vs 8.8%, *p* < 0.05) and that educational materials on food allergy were available in the lunchroom (14.5% vs 4%, *p* < 0.01).

**Table 4:** Unadjusted Association with Outcomes: Public vs. Private (Yes vs. No/Unsure/No Response)

Policy	Type of School	
	Private	Public
	*N* = 62	*N* = 227
School is a generally safe environment	44 (71.0)	166 (73.1)
Epinephrine Policies		
Emergency (stock) epinephrine is available	29 (46.8)	126 (55.1)
Children are able to carry their medications	37 (59.7)	130 (57.3)
Child’s epinephrine is readily available in the classroom	39 (62.9)	106 (46.7)*
Emergency (stock) epinephrine available on all school field trips	25 (40.3)	79 (34.8)
Emergency (stock) epinephrine available for after-school activities	9 (14.5)	21 (9.3)
Emergency (stock) epinephrine travels with groups outside of school	9 (14.5)	24 (10.6)
Lunchroom Policies		
Designated lunch areas for students with food allergies	31 (50.0)	153 (67.4)*
School lunch menus with allergen information available	18 (29.0)	82 (36.1)
Food items are labeled with allergen information	9 (14.5)	27 (11.9)
Clear cleaning procedures in the lunchroom	37 (59.7)	123 (54.2)
Classroom Policies		
Snack policy in the classroom	36 (58.1)	142 (62.6)
Strict food guidelines for celebrations (holidays and birthdays)	27 (43.6)	126 (55.5)
Field Trip & After-School Policies		
When food is not provided by the school for field trips, all parents are provided with food guidelines	24 (38.7)	50 (22.0)**
Strict food policies for after-school activities	8 (12.9)	15 (6.6)
Concessions are clearly labeled for food allergens	4 (6.5)	14 (6.2))
Food Allergy Education Policies		
Training and education for students	11 (17.7)	20 (8.8)*
Educational materials in the lunchroom relating to food allergy	9 (14.5)	9 (4.0)**
Educational materials in the classroom relating to food allergy	5 (8.1)	11 (4.9)
Children take school bus to/from school	17 (27.4)	112 (49.3)
Adult on school bus is trained on allergic reactions	6 (35.3)	42 (37.5)

** *p* < 0.01, * *p* < 0.05

Differences in food allergy policy were also identified based on geographic location (Table 5). Parents in the Northeast more frequently reported that food items in the lunchroom were labeled with allergen information as compared to other regions (NE = 19.6%, MW = 9.5%, South = 3.2%, West = 0%, *p* < 0.05). Parents in the Northeast and Midwest more frequently reported strict food allergy guidelines for classroom celebrations as compared to those in the South and West (NE = 59.8%, MW = 56.2%, South = 35.5%, West = 30.0%, *p* < 0.05). Parents of students who rode the school bus to/from school in the South more frequently reported that there is an adult present on the school bus who was trained in the management of allergic reactions as compared to parents in the Northeast, Midwest, and West (South = 81.3%, NE = 24.6%, MW = 43.3%, West = 25%; *p* < 0.01).

**Table 5:** Unadjusted Association with Outcomes: Region (Yes vs. No/Unsure/No Response)

Policy	Region				
	Northeast	Midwest	South	West	Unknown
	*N* = 102	*N* = 105	*N* = 31	*N* = 20	N = 31
School is generally safe environment	82 (80.4)	75 (71.4)	22 (71.0)	11 (55.0)	20 (64.5)
Epinephrine Policies					
Emergency (stock) epinephrine is available	54 (52.9)	59 (56.2)	17 (54.8)	8 (40.0)	17 (54.8)
Children are able to carry their medications	52 (51.0)	65 (61.9)	17 (54.8)	13 (65.0)	20 (64.5)
Child’s epinephrine is readily available in the classroom	46 (45.1)	57 (54.3)	18 (58.1)	7 (35.0)	17 (54.8)
Emergency (stock) epinephrine available on all school field trips	34 (33.3)	42 (40.0)	12 (38.7)	2 (10.0)	14 (45.2)
Emergency (stock) epinephrine available for after-school activities	9 (8.82)	16 (15.2)	3 (9.7)	1 (5.0)	1 (3.2)
Emergency (stock) epinephrine travels with groups outside of school	13 (12.8)	11 (10.5)	1 (3.2)	1 (5.0)	7 (22.6)
Lunchroom Policies					
Designated lunch areas for students with food allergies	63 (61.8)	74 (70.5)	20 (64.5)	8 (40.0)	19 (61.3)
School lunch menus with allergen information available	36 (35.3)	28 (26.7)	14 (45.2)	9 (45.0)	13 (41.9)
Food items are labeled with allergen information	20 (19.6)	10 (9.5)	1 (3.2)	0 (0.0)	5 (16.1)*
Clear cleaning procedures in the lunchroom	60 (58.8)	57 (54.3)	18 (58.1)	9 (45.0)	16 (51.6)
Classroom Policies					
Snack policy in the classroom	64 (62.8)	70 (66.7)	20 (64.5)	8 (40.0)	16 (51.6)
Strict food guidelines for celebrations (holidays and birthdays)	61 (59.8)	59 (56.2)	11 (35.5)	6 (30.0)	16 (51.6)*
Field Trip & After-School Policies					
When food is not provided by the school for field trips, all parents are provided with food guidelines	30 (29.4)	24 (22.9)	6 (16.1)	4 (20.0)	11 (35.5)
Strict food policies for after-school activities	8 (7.8)	11 (10.5)	0 (0.0)	1 (5.0)	3 (9.7)
Concessions are clearly labeled for food allergens	6 (5.9)	6 (5.7)	0 (0.0)	2 (10.0)	4 (12.9)
Food Allergy Education Policies					
Training and education for students	10 (9.8)	13 (12.4)	3 (9.7)	2 (10.0)	3 (9.7)
Educational materials in the lunchroom relating to food allergy	5 (4.9)	9 (8.6)	2 (6.5)	1 (5.0)	1 (3.2)
Educational materials in the classroom relating to food allergy	5 (4.9)	4 (3.8)	3 (9.7)	2 (10.0)	2 (6.45)
Children take school bus to/from school	65 (63.7)	30 (28.6)	16 (51.6)	4 (20.0)	14 (45.2)
Adult on school bus is trained on allergic reactions	16 (24.6)	13 (43.3)	13 (81.3)	1 (25.0)	5 (35.7)**

**p < 0.01, *p < 0.05

## Discussion

The health and safety needs of students must be met so that children can thrive and achieve their academic potential in a safe and inclusive environment. To date, given the lack of research on the most effective strategies to manage food allergy in the school setting and the subsequent lack of standardized national and local requirements, schools use a variety of approaches to manage food allergy and to minimize the risk of accidental exposures to food allergens. To our knowledge, the present study was one of the first to report on school food allergy polices from the perspective of parents of children with food allergy. Importantly, approximately one in five parents in our study did not feel that their food allergic child was safe while at school. Significant variations were reported in food allergy management and anaphylaxis preparedness strategies and appeared to be affected by the age of the student body, type of school (public versus private), and geographic location. Additionally, while the majority of parents felt that the polices in place in their child’s school were helpful, most also believed that the implementation of additional polices was necessary, including policies related to epinephrine access, labeling of food items, and food allergy education and training.

Half of the parents in our study reported that their child’s school carried non-student-specific stock epinephrine, with an additional one-quarter being unaware of whether their child’s school had stock epinephrine available. Over 90% of parents felt that this policy was either helpful or needed. Whereas most states have legislation allowing schools to voluntarily stock undesignated epinephrine auto-injectors (EAI), few states have legislative mandates requiring that schools do so [11, 12]. In states without a mandate, barriers to stock epinephrine availability may include administrative and staff resistance, lack of adequate staff education, and cost [13–15]. However, given that prompt administration of epinephrine is the only life-saving treatment for anaphylaxis and that 25% of cases of anaphylaxis in schools occur in children previously undiagnosed with a food allergy [9, 16, 17], improving the availability of stock epinephrine should be a priority in improving the management of food allergy in the school setting. The majority of parents also desired that stock epinephrine be available on school field trips and during after-school activities. Such policies may pose a challenge for schools, as they require the availability of additional EAIs. However, as up to 19% of anaphylactic reactions during the school day occur outside the school building or on field trips, the availability of stock EAIs for these situations is an important measure to consider [18].

Several policies were infrequently reported to be in place, but frequently deemed to be needed. Half of parents reported that food items sold at lunch and concessions after-school were not labeled with allergen information, though over 80% felt that such labeling should be implemented. Similarly, only 44% of parents indicated that lunch menus with allergen information were available to them, with 85% feeling that this policy was needed. Because thorough review of ingredients in all food and drink products prior to consumption is a core strategy for food allergen avoidance and anaphylaxis prevention [19] widespread implementation of ingredient labeling policies should be prioritized in order to protect students and prevent potential allergic reactions at school. Similarly, policies related to food allergy training and education (i.e., student education, materials available in the lunchroom and classroom, and training of school bus staff) were among those least frequently reported to be in place, though approximately four in five parents felt that such policies should to be implemented. Educational programs have been shown to be effective in increasing food allergy knowledge as well as appropriate use of an EAI and may be an additional key area of policy on which to focus [20, 21].

Expectedly, several school food allergy polices appeared to be driven by the age of the student body. For instance, parents of elementary and middle school students more frequently reported designated lunch areas and food allergy policies for classroom snacks and celebrations compared to parents of high school students. These differences are likely age-appropriate, as younger children are less developmentally and cognitively ready to self-manage and to minimize their risk of accidental food allergen ingestions. Similarly, middle and high school students were more frequently reported to be allowed to self-carry epinephrine, which is consistent with greater autonomy and food allergy self-management skills with age. Importantly, adolescents remain at greatest risk of poor outcomes from food allergy [22–24]. This unique population may therefore particularly benefit from increased training and education on food allergy, a policy which was desired by the majority of survey respondents.

Differences in school policy also appeared to exist between public and private schools. Private schools provided their students with more food allergy training and education, had stricter food guidelines for field trips when food was not provided by the school, and were more likely to have epinephrine available in the classroom. These differences may be based on variations in financial and time resources, staffing, and level of school nurse coverage. Parents of children with food allergy may also have more influence in shaping policy decisions at private as compared to public schools.

Although the majority of parents felt that their child’s school was generally safe, one in four were not sure or did not consider the school environment to be safe for their food allergic child. Such anxiety about safety and the potential for allergic reactions may negatively impact quality of life for students and their families and adversely affect school attendance [25, 26]. Further study is warranted to investigate the reasons behind these negative perceptions and potential opportunities for improvement. For example, clear documentation of student-specific medical needs with medical forms (e.g. individualized health plan, anaphylaxis emergency plan, school 504 plan), clear labeling of food items sold at school, an adequate supply of appropriate medications (including stock epinephrine), and food allergy/anaphylaxis education for school staff may improve parental perceptions of safety during the school day [4, 27–29].

It is also notable that parents were frequently unaware of whether certain food allergy policies were in place in their child’s school. For instance, 40-50% were unsure about the availability of stock epinephrine for after-school activities and approximately 40% were uncertain of whether food items sold at lunch or after school were labeled with allergen information. It is possible that many parents were not affected by such policies (e.g., children brought their own food to school or did not participate in after-school activities). However, clear and timely communication and a collaborative approach between the student’s school and family may provide an opportunity to help parents be more aware about policies that are in place and therefore feel that school is a safer environment for their food allergic child [30].

Several limitations of our study should be mentioned. The use of self-report in data collection includes inherent risk to internal validity related to inaccurate recall bias, selective recall bias and social desirability bias. Additionally, respondents were recruited through food allergy support and advocacy organizations and were predominantly Caucasian, college-educated, and high-income individuals. Additionally, the survey was distributed exclusively online, in English. Such limitations suggest that these findings may not be generalizable to the broader U.S. population of families with food allergies. For example, parents with higher household income may be more likely to have children at schools in good financial standing and with more resources available for food allergy management. Future efforts should be made to include underrepresented groups from a more diverse socioeconomic background, in hopes of having a more representative population. Lastly, given the nature of the survey, our results are undoubtedly affected by the potential lack of parental awareness regarding specific food allergy policies. This lack of awareness, however, is also a notable finding.

## Conclusion

This study demonstrates that while most schools reportedly have one or more food allergy policies in place, a substantial proportion of parents have concerns over the safety of their child in the school setting. Many parents feel that additional policies are necessary to improve the safety of the school environment for children with food allergy. The availability of stock epinephrine, improved allergen labeling of food and menus provided by the school and increased food allergy education may be key areas of policy on which to focus. Additionally, many parents may not be aware of all the policies that are already in place in their child’s school, which highlights the importance of maintaining an ongoing dialogue between parents, school administration, school nurses, pediatric health care providers, and school staff. There also appears to be differences in food allergy policies related to student age, school type, and geographic location. Future studies may investigate the reasons behind such policy variations and identify those policies that are most effective in creating a safe school environment, thus promoting better clinical outcomes for children with food allergy in the school setting.

## Abbreviation

EAI: Epinephrine auto-injector
